# Adenovirus 40 and 41 Antibodies Associated With Protection From Infection in a Bangladeshi Birth Cohort

**DOI:** 10.1093/infdis/jiaf558

**Published:** 2025-11-05

**Authors:** Jennifer Hendrick, Jennie Z Ma, Vu Huynh, Jozelyn V Pablo, Andy A Teng, Amit Oberai, Joseph J Campo, David Camerini, William A Petri

**Affiliations:** Department of Medicine, University of Virginia Health System, Charlottesville, Virginia, USA; Department of Public Health Sciences, University of Virginia School of Medicine, Charlottesville, Virginia, USA; Antigen Discovery Incorporated (ADI), Irvine California, USA; Antigen Discovery Incorporated (ADI), Irvine California, USA; Antigen Discovery Incorporated (ADI), Irvine California, USA; Antigen Discovery Incorporated (ADI), Irvine California, USA; Antigen Discovery Incorporated (ADI), Irvine California, USA; Antigen Discovery Incorporated (ADI), Irvine California, USA; Center for Virus Research, University of California, Irvine, California, USA; Department of Developmental and Cell Biology, University of California, Irvine, California, USA; Department of Medicine, University of Virginia Health System, Charlottesville, Virginia, USA; Department of Microbiology, Immunology and Cancer Biology, University of Virginia Health System, Charlottesville, Virginia, USA

**Keywords:** adenovirus 40/41, antibody response, pediatric gastroenteritis

## Abstract

**Background:**

Adenovirus (AdV) 40/41 is a major cause of pediatric acute gastroenteritis (AGE), leading to significant morbidity and mortality worldwide. As little is known about clinical correlates of protection, we analyzed AdV-specific antibody reactivities using a multi-AdV protein microarray and serum from a Bangladeshi birth cohort surveilled for diarrhea during the first 2 years of life.

**Methods:**

Arrays contained a comprehensive set of proteins from AdV 40 and 41, in addition to respiratory AdVs 4, 5, and 26. Children were split into four groups according to AdV 40/41 infection occurrence during year 1 (Y1) and year 2 (Y2) of life. One-year array antibody reactivity levels were analyzed from 119 children using principal component analysis (PCA). Top antibody reactivities were evaluated for associations with AdV 40/41 disease severity and protection in Year 2 using logistic regression.

**Results:**

Eight principal components (PCs) were identified from PCA. Top targets contributing to the leading PCs included external AdV 40/41 antigens that function in host cell entry, particularly penton base (PB) proteins. AdV 41 PB antibody reactivity at Year 1 is significantly associated with reduced risk of AdV 40/41 infection in Year 2 (OR = 0.36, 95% CI: 0.16–0.80, *P* = .013, adjusted *P* = .003). Additionally, those with mild to moderate infections in Year 2 had higher reactivities to AdV 40 and 41 PB compared to severe infections (*P* = .008 and .032, respectively).

**Conclusions:**

Higher antibody reactivity to AdV PB was associated with improved Year 2 AdV 40/41 outcomes, elevating it as a promising vaccine or monoclonal antibody target.

## Teaser

In a Bangladeshi birth cohort, Year 1 antibody reactivity targeting the external adenovirus 40/41 penton base (PB) protein was associated with reduced Year 2 adenovirus 40/41 infection and severity, making it a promising target for vaccine or monoclonal antibody development.

Although in recent decades we have seen steady improvement in diarrheal disease-related mortality and, as a result, regional life expectancy in key areas like sub–Saharan Africa and South Asia, enteric infections remain a leading cause of morbidity and mortality in children under 5 [1–3]. Analysis of the Global Enteric Multicenter Study (GEMS), a large multi-country case control study of childhood diarrhea, using broad range quantitative polymerase chain reaction (qPCR) revealed the two closely related adenoviruses, 40 and 41 (AdV 40/41), as the third highest cause of diarrheal illness in children under 2 years of age [4]. The World Health Organization (WHO)-coordinated Global Rotavirus Surveillance Network further identified AdV 40/41 as the leading cause of hospitalized diarrhea in India in children under age 5 [5, 6]. This was also seen in Sub-Saharan Africa sites in the Vaccine Impact on Diarrhea in Africa (VIDA) study where rotavirus and AdV 40/41 acute gastroenteritis episodes (AGE) were found to be the most severe [7].

Adenoviruses are delineated into 7 species (A to G) with Species F containing the serotypes F40 and F41, which have a pronounced tropism for the gastrointestinal (GI) tract [8]. Although additional AdV serotypes have been linked to enteric disease [9–11], serotypes 40 and 41 are most associated with AGE [12]. Multiple cohort studies in Bangladesh, including The Performance of Rotavirus and Oral Polio Vaccines in Developing Countries (PROVIDE) study (enrolled from 2011 until 2013) and a subsequent longitudinal cohort study (Dhaka Birth Cohort, enrolled from 2014 until 2016), have identified AdV 40/41 as a leading cause of diarrheal illness in children [2, 3, 13, 14]. In both the PROVIDE and Dhaka Birth Cohorts [2, 3], many children were repeatedly infected with AdV 40/41 in the first few years of life, suggesting inefficient and/or transient immune response to initial infection.

Adenoviruses are non-enveloped, double-stranded DNA viruses with an icosahedral capsid, the majority of which is made up of hexons [8]. At each of the 12 capsid vertices are penton bases (PB) that anchor the N-terminal tails of protruding trimeric fibers. This allows for a two-step entry process into host cells: interaction of the fiber proteins with cellular receptors then interaction of the PB with integrin co-receptors [15]. One of the major known differences between non-enteric and enteric AdVs is the two different types of fiber proteins, one long and one short, allowing for resistance to low pH environments and host cell attachment. Additionally, while all other AdVs contain a conserved RGD motif (Arg-Gly-Asp) in the PB, both AdV 40 and 41 lack this sequence and likely utilize different integrins as co-receptors for cell entry [8, 15, 16]. These structures may generate specific, protective, neutralizing antibodies to AdV 40/41 through limiting key interactions with the host cell.

Among the five enteric viruses known as major contributors to diarrheal infection, rotavirus is the only one with an available vaccine. Outside of neutralizing seroprevalence studies [17, 18], there is little known about the character and efficacy of active antibody immunity to AdV 40/41, a crucial first step to vaccine development. To begin to address this knowledge gap, we used a multi-adenovirus microarray and year 1 serum from children in the Dhaka Birth Cohort to determine top antibody reactivities associated with reduced year 2 AdV 40/41 infection.

## METHODS

### Cohort Characteristics and Sample Collection

The Dhaka Birth Cohort enrolled mothers and infants from June 2014 to March 2016 in Mirpur, Dhaka, Bangladesh. Infants were enrolled by day 7 of life. In-home diarrheal surveillance was conducted twice weekly, and stool was collected if caregivers reported >3 unformed stools in 24 hours, with ≥3 days since a prior episode. Child blood was collected at multiple timepoints during the first 2 years. Samples were stored at 4°C in the field and transported to the International Centre for Diarrhoeal Disease Research, Bangladesh (icddr,b).

### Stool Testing and Interpretation

Diarrheal stool samples were tested for pathogen carriage using a TaqMan Array Card (TAC) on a subset of cohort children (n = 219). TAC is a qPCR platform capable of detecting 36 enteric pathogens via previously described methods [19]. AdV 40/41 detection on this panel targets the long fiber gene specific to these serotypes. Episode-specific attributable fractions (AFes) were calculated for each pathogen per diarrheal episode based on qPCR cycle threshold (CT) values, as described in GEMS [4]. Briefly, in GEMS, odds ratios (ORs) were derived from a multivariable conditional logistic regression model that related pathogen quantity to diarrhea while adjusting for co-detected pathogens. In this Dhaka Birth Cohort subset, AFes were calculated using the original GEMS model coefficients, providing an estimate of each pathogen's relative contribution to illness. An AFe threshold of 0.5 or higher [3, 7] was used to indicate that the detected pathogen was a significant contributor to the diarrheal episode.

### Sample Selection and Data Collection

**Figure 1.**
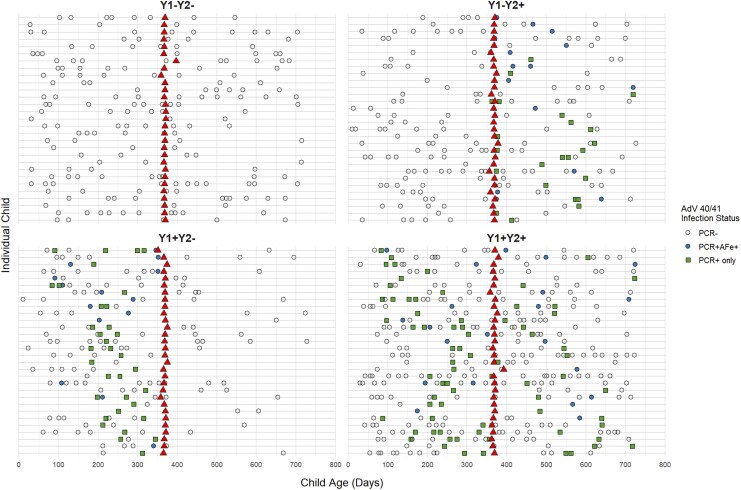
Diarrheal episodes and serum collections by Adenovirus (AdV) 40/41 infection group. Each horizontal gray line represents an individual child's timeline with x-axis displaying their age in days. Each facet shows children grouped by year 1 (Y1) and y 2 (Y2) infection status. Points indicate diarrheal episodes: green-filled squares are AdV 40/41 PCR-positive episodes (cycle threshold [CT] < 35); blue-filled circles represent AdV 40/41 attributable fraction (AF) positive episodes (AFe ≥ 0.5); open circles are AdV 40/41 PCR-negative episodes. Red triangles mark serum sample collection times.

One-year serum samples from 119 children were selected for microarray analysis based on AdV 40/41 infection status: year 1 only (Y1 + Y2-, n = 30), year 2 only (Y1-Y2+, n = 30), both years (Y1 + Y2+, n = 30), or no detected infection (Y1-Y2-, n = 29) (Figure 1**).** The one-year sample timepoint was chosen to adequately capture reinfections while minimizing the influence of maternally derived antibodies, which have largely waned by this age [20, 21]. For children with multiple AdV 40/41 PCR-positive episodes within the year, the episode with the lowest CT (highest viral burden) was selected. Those children without any PCR reactivity from stool samples collected throughout the year were considered negative for that year. Child temperature, episode duration, diarrhea frequency, additional symptoms, and clinical course (hospitalizations, need for fluids, antibiotics, etc.) were collected for each episode.

### Multi Adenovirus Microarray Synthesis and Probing

Human adenovirus types 4, 5, 26, 40, and 41 genomes as well as the chimpanzee adenovirus Y25 genome were obtained from the American Type Culture Collection (ATCC) and used as templates for synthesis of mature viral proteins. Over 70 AdV 40 and 41 proteins were synthesized, including hexon, PB, short fiber (L5), long fiber, protein IX, protein IIIa, protein VIII, protein VI, core, and enzyme proteins [8]. The array also contained all proteins generated from AdV 4, 5, 26, and Y25, which cause upper respiratory illness in humans and were utilized as vectors in COVID-19 vaccines (AdV 5, 26, and Y25) [22].

DNA or cDNA encoding each viral protein was PCR-amplified and inserted into the vector pXT7 by recombination in *Escherichia coli (E. coli)* to enlarge an established library of AdV coding sequences. After clone quality check, all proteins were expressed using a coupled *E. coli* cell-free in vitro transcription and translation (IVTT) system and spotted onto nitrocellulose-coated slides. Expression of spotted IVTT proteins was verified by probing arrays with monoclonal antibodies against the His and HA tags present on each protein. Each array also included controls for both systematic and biological effects. Human immunoglobulins (Ig) were spotted at multiple dilutions on each subarray to monitor secondary antibody reactivity, while anti-human immunoglobulin monoclonal antibodies were included to capture IgG, IgA, and IgM as probing controls. IVTT spots lacking target protein DNA were also included to assess biological cross-reactivity and provide normalization factors.

Serum was used to probe the array following a 1:100 dilution. After overnight incubation, bound IgG was detected with Bethyl DyLight 650-conjugated goat anti-human IgG-Fc (Fortis Life Sciences, Cat#A80-104D5, Boston, MA, USA). Chips were scanned and quantified using an InnoScan 910AL microarray scanner (Innopsys, Carbonne, France).

The expression and antigenicity of the AdV antigens on this array were previously established; they were included in a prior study on a multipathogen protein microarray used to assess antibodies in human milk and serum from a large cohort across a wide range of pathogens [23]. Arrays from the same platform have previously demonstrated antibody signal intensities that correlated strongly with ELISA titers [24].

### Initial Data Cleaning

This study focused on serum immunoglobulin (Ig) G reactivities. Array data were normalized to remove systematic effects by subtracting the median Log_2_ signal intensity of the IVTT control spots for each sample. Normalized protein data were used to classify antigens as “reactive” if they were seropositive in greater than 10% of the cohort. A seropositivity threshold was established as a normalized signal intensity (SI) of 1.0, or twice the IVTT control signals on the Log_2_ scale. Antibody magnitude was assessed as normalized SI.

### Statistical Analysis

The primary objective of this analysis was to identify the top AdV 40/41 antibody responses associated with protection against year 2 AdV 40/41 infection. Secondary analyses included evaluating the associations between these top antibody reactivities and infection burden, disease severity, and coinfections, as well as the correlations between antibody reactivities across AdV serotypes. All analyses were conducted in SAS (v9.4), with random forest analyses and plots generated in R (v4.3).

### Determine Infection Group Characteristic Differences and Select Model Covariates

To characterize potential confounders, we compared child, maternal, and household characteristics across the four infection groups (Y1 + Y2−, Y1 + Y2+, Y1−Y2+, Y1–Y2–). Continuous variables were compared using the Wilcoxon rank-sum (two groups) or Kruskal–Wallis tests (>2 groups), and categorical variables with Chi-square or Fisher's exact tests when expected cell counts were small. Covariates for regression models were selected based on observed significant differences across infection groups and their potential to confound associations between year 1 antibody reactivity (exposure) and year 2 infection occurrence (outcome). Pairwise correlations among all clinical characteristics were evaluated using Spearman rank correlation (*P* < .05 and |ρ|≥0.7) to assess collinearity and ensure model stability (Supplementary Figure 1**).**

### Dimensionality Reduction to Identify Antibody Patterns Associated With Year 2 Infection

Given the large number of correlated antibody measurements, analyzing each individually would raise concerns about multiple testing. To address this, the analysis was conducted in two steps. First, principal component analysis (PCA) was applied for dimensionality reduction to summarize correlated antibody patterns. Because the antibody data were skewed, reactivities were ranked within the cohort before applying parametric PCA. Leading principal components (PCs) were selected based on a combination of the scree plot (elbow method) and eigenvalues greater than 1. Second, these leading PCs were tested for association with year 2 infection using logistic regression, adjusting for year 1 infection status and previously selected covariates. The PCs with the lowest *P*-values and smallest odds ratios, indicating the strongest association with reduced odds of year 2 infection, were selected to identify their top-loading antibodies (those contributing most strongly to the component) for further evaluation.

### Association of Top Antibody Reactivities With Year 2 Infection

Top AdV 40/41 antibody reactivities identified through PCA were first assessed for differences across infection groups (Y1 + Y2–, Y1 + Y2+, Y1–Y2+, Y1–Y2–) using Kruskal–Wallis tests. Reactivities were then categorized into tertiles due to skewed distributions (Supplementary Figure 2) and analyzed for association with year 2 infection using logistic regression, allowing estimation of both the direction and magnitude of effect. All models were adjusted for year 1 infection status, and the final model included previously selected covariates to account for potential confounding.

To complement the regression-based approach, a random forest (RF) algorithm was applied to evaluate nonlinear associations between antibody reactivities and year 2 infection. Since RF can deal with large yet potentially correlated predictors, all unranked AdV antibody reactivities from the array were assessed simultaneously in the RF analysis. Optimal tuning parameters, including the number of trees and predictors per split, were determined via grid search with 10-fold cross-validation using the caret package in R. Variable importance was assessed using mean decrease in Gini index (randomForest package in R) and visualized for the top contributors.

### Association of Top Antibody Reactivities With Year 2 Disease Severity and Viral Burden

Ruuska scores, a validated composite measure of diarrhea and vomiting frequency and duration, temperature, dehydration, and treatment, were calculated for all AdV 40/41 episodes. Differences in antibody reactivities across children with mild, moderate, and severe year 2 infections (as determined by Ruuska scoring) were analyzed using Kruskal–Wallis tests. Among children with year 2 AdV 40/41 infection, viral load (quantified by cycle threshold [CT] and episode-specific attributable fraction [AFe]) was modeled as a continuous outcome using linear regression, with top antibody reactivities included as predictors. Because this analysis was restricted to children with year 2 infection, models were adjusted only for year 1 infection status to ensure valid estimation and avoid overfitting given the smaller sample size.

### Correlation Between Antibody Reactivities Across AdV Serotypes

Given the skewed distributions of antibody reactivities, Spearman correlation was used to evaluate pairwise relationships among external capsid antibodies across all AdV serotypes on the array (AdV 4, 5, 26, 40, and 41).

### Common AdV 40/41 Coinfections and Top Antibody Associations

For each diarrheal episode, coinfection status was defined as PCR-positive AdV 40/41 together with at least one additional enteric pathogen with AFe ≥ 0.5. A child was considered to have experienced a coinfection if any AdV 40/41 episode met this criterion during the first two years of follow-up. The proportion of AdV 40/41 episodes with ≥1 co-infection was compared across infection groups using Chi-square tests. Top AdV 40/41 antibody reactivities were then compared between children who had ever experienced a coinfection versus those who did not using two-sided Wilcoxon rank-sum tests.

## RESULTS

One hundred and nineteen children were classified into four groups based on AdV 40/41 infection status in Year 1 and Year 2 (Figure 1). Among infection groups, child enrollment height for age Z score (HAZ) was highest among those without detectable AdV 40/41 infection in the first 2 years (*P* = .02, Table 1). Year 2 reinfections were more common in male children (*P* = .02). Other child and maternal demographics, including breastfeeding, did not differ between infection groups. Children from households sharing toilets were more likely to have no infections or only Year 1 infections (*P* = .02), while other household characteristics were similar across groups (Table 1).

**Table 1.** Bangladeshi Birth Cohort Characteristics by Adenovirus (AdV) 40/41 Year 1 (Y1) and Year 2 (Y2) Infection Status. Continuous Variables are Expressed as Median (IQR). Categorical Variables are Expressed as n (%) of the Infection Group. P-values Listed in the Last Column are Results From Chi Square Test and Kruskal-Wallis Test Between All Four Infection Groups for Categorical and Continuous Variables, Respectively. Y1+: Positive for AdV 40/41 Infection During Year 1 of Life; Y1-: Negative for AdV 40/41 Infection During Year 1 of Life; Y2+: Positive for AdV 40/41 Infection During Year 2 of Life; Y2-: Negative for AdV 40/41 Infection During Year 2 of Life; HAZ: Height-for-Age Z-score; WAZ: Weight-for-Age Z-score; H.S.C.: Higher Secondary Certificate (Completed and Passed Exam in the 12th Year of School); S.C.C.: Secondary School Certificate (Completed and Passed Exam in the 10th Year of School). *Other 2 Children Fed Top Milk (Packaged, Heat-treated Cow's Milk) ^Other Children Fed Water/Sugar or Water/Honey Mixture

	Total N = 119	Y1 + Y2- N = 30	Y1 + Y2+ N = 30	Y1-Y2+ N = 30	Y1-Y2- N = 29	*P V*alue
Child						
Age upon Enrollment (d)	3 (2)	3 (3)	3 (3)	3.5 (3)	2 (2)	.06
Male sex	50 (42)	14 (47)	19 (63)	8 (27)	9 (31)	.02
Enrollment HAZ	−1.15 (1.27)	−1.34 (0.8)	−0.93 (1.26)	−1.17 (1.24)	−0.79 (1.57)	.02
Enrollment WAZ	−1.25 (1.56)	−1.83 (1.54)	−0.97 (1.33)	−1.08 (1.66)	−1.15 (1.07)	0.14
1 Year HAZ	−1.13 (1.37)	−1.07 (1.41)	−1.21 (1.74)	−1.04 (1.94)	−1.36 (1.03)	.17
1 Year WAZ	−1.43 (1.42)	−1.72 (0.92)	−1.63 (1.57)	−1.25 (1.45)	−1.06 (1.19)	.96
Breastfed at Enrollment	111 (93)	28 (93)^	28 (93)*	28 (93)*	27 (93)^	.99
Mother						
Age upon Enrollment (y)	23 (6)	22 (6)	22.5 (9)	23 (6)	24 (5)	.76
BMI upon Enrollment	22.6 (5.2)	22.87 (6.3)	23.6 (6.6)	22.6 (5.4)	21.8 (3.1)	.35
Education Level	…	…	…	…	…	.29
H.S.C. Passed	2 (3)	3 (10)	1 (3)	0 (0)	2 (7)	
S.S.C. Passed	4 (7)	2 (7)	2 (7)	2 (7)	2 (7)	
Some Secondary	14 (24)	7 (23)	7 (23)	10 (33)	4 (14)	
Some Primary	27 (46)	11 (37)	13 (43)	14 (47)	13 (45)	
None	12 (20)	7 (23)	4 (13)	4 (13)	8 (28)	
Occupation	…	…	…	…	…	.83
Home Caretaker	99 (83)	26 (87)	25 (83)	25 (83)	23 (79)	
Student	2 (2)	0 (0)	0 (0)	1 (3)	1 (3)	
Teacher	1 (1)	0 (0)	1 (3)	0 (0)	0 (0)	
Textile Industry	15 (13)	4 (13)	3 (10)	4 (13)	4 (14)	
Business Owner	2 (2)	0 (0)	1 (3)	0 (0)	1 (3)	
Household						
Total Yearly Income (taka)	14 000 (10 000)	12 500 (10 000)	12 500 (7500)	16 000 (12 000)	14 000 (8000)	.38
Number of Rooms	1 (1)	1 (1)	2 (1)	1 (2)	1 (1)	.79
Number of Children	2 (1)	1.5 (1)	2 (2)	1 (1)	2 (2)	.65
Number of Siblings Under 5	0 (1)	0 (0)	0 (0)	0 (1)	0 (1)	.92
Open Drain Beside House	40 (33)	10 (33)	11 (37)	10 (33)	9 (31)	.98
Toilet Shared with Other Households	79 (66)	24 (80)	17 (57)	15 (50)	23 (79)	.02
Boiled or Treated Household Water	87 (73)	20 (67)	20 (67)	25 (83)	22 (76)	.35

PCA was conducted on all AdV targets on the microarray, and eight leading PCs were identified (Supplementary Table 1), accounting for 69% of the total variation. Of these leading PCs, PC2, which explained 11.7% of the total variation, showed the strongest association with year 2 infection in the logistic regression when adjusting for the occurrence of Year 1 infection (OR = 0.72, CI: 0.49, 1.05, *P* = .084, Supplementary Figure 3). After further adjusting for sex, child enrollment HAZ, and household shared toilet, a higher PC2 score was associated with a reduced risk of AdV 40/41 infection in Year 2 (OR = 0.65, CI: 0.43, .98, *P* = .039, Supplementary Figure 3).

**Figure 2.**
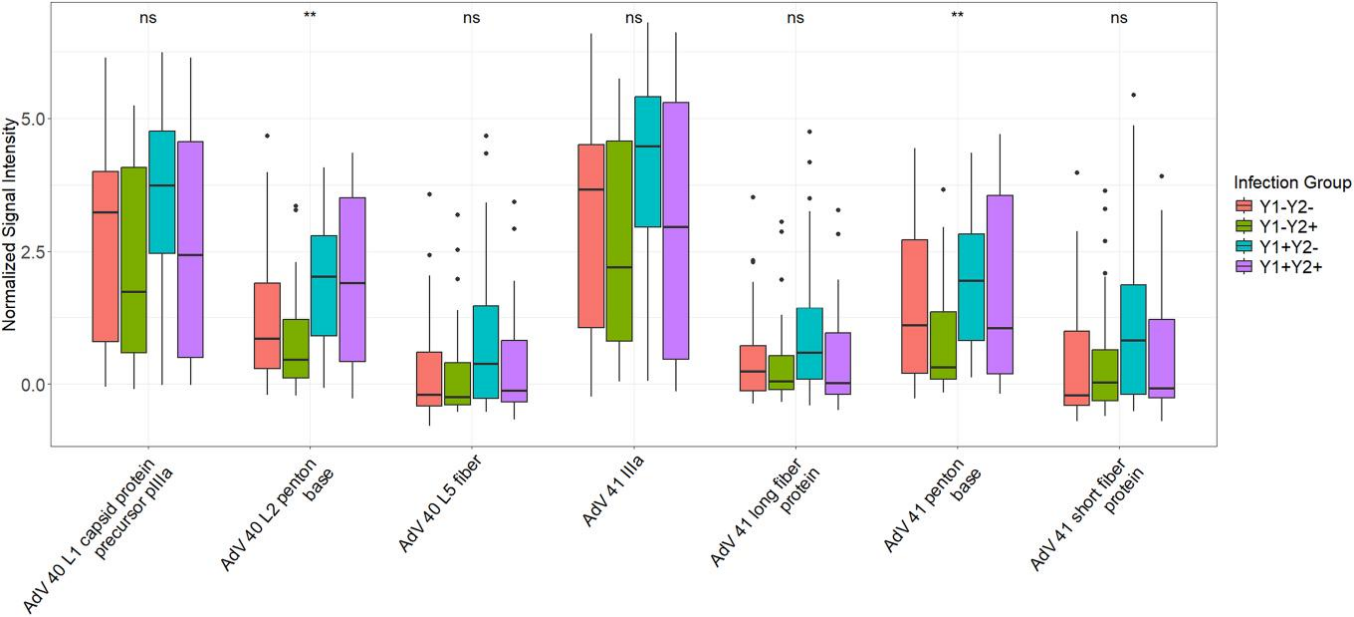
Box and whisker plots of top Adenovirus (AdV) 40 and 41 antigen reactivities in principal component (PC) 2 by infection group. NS = non-significant *P* value, * = *P* ≤ .05, and ** = *P* ≤ .01 by Kruskal–Wallis test. Y1+: Positive for AdV 40/41 infection during y 1 of life; Y1-: Negative for AdV 40/41 infection during y 1 of life; Y2+: Positive for AdV 40/41 infection during y 2 of life; Y2-: Negative for AdV 40/41 infection during y 2 of life.

Top AdV 40/41 targets loaded into PC2 included the external antigens: AdV 40 and 41 penton base (PB) proteins, AdV 40 and 41 short fiber protein, and AdV 41 long fiber protein **(**Supplementary Table 1, Supplementary Figure 4). Of the top AdV 40/41 reactivities in PC2, AdV 40 and 41 PB reactivities were significantly different among all four infection groups (*P* = .007 and *P* = .002, respectively, Figure 2).

**Figure 3.**
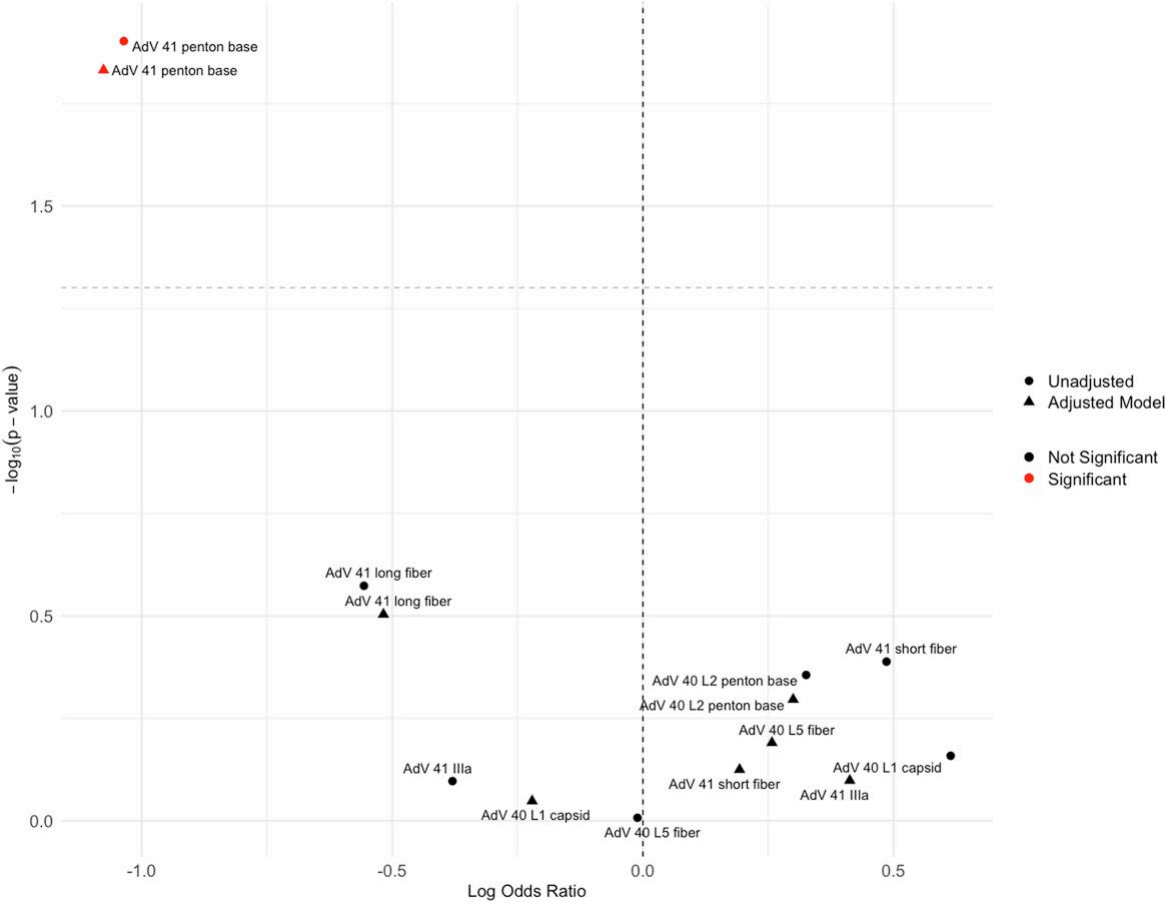
Volcano plot of the top 7 AdV 40/41 antibodies showing associations with year 2 AdV 40/41 infection occurrence. Shapes indicate model type (circle = unadjusted, triangle = covariate-adjusted), and colors indicate significance (red = *P* < .05, black = *P* ≥ .05). The y-axis is −log10(*P*-value) and the x-axis is log odds ratio. Dashed lines indicate nominal significance (horizontal) and null effect (vertical).

These top-ranked AdV 40/41 antigen reactivities from PC2 were then individually analyzed for their association with year 2 AdV infection. AdV 41 PB was the only antibody reactivity significantly associated with a reduced risk of year 2 infection (OR = 0.36, 95% CI: 0.16, .80, *P* = .013) (Figure 3). This association remained significant (OR = 0.45, 95% CI: 0.27, .76, *P* = .003) after adjusting for sex, child enrollment HAZ, household shared toilet, and year 1 infection status (Supplementary Figure 5). As a complementary approach, a random forest analysis of all AdV antigen reactivities from the array revealed AdV 41 PB as the most important variable associated with year 2 infection **(**Supplementary Figure 6**).**

Among those infected, AdV 40/41 episode characteristics between the three infection groups (Y1+, Y2-, Y1+,Y2 + and Y1-, Y2+) are summarized in Table 2. Ruuska scores were lower for year 2 infections compared to those in year 1 (*P* = .047), with more moderate (RUUSKA 7–10) and severe infections (Ruuska ≥ 11) occurring in year 1 (*P* = .042, Table 2). There were no statistically significant differences in Ruuska scores or in the individual clinical components of the score between these infection groups. Year 1 AFe and CT values did not differ statistically between children with or without year 2 reinfection, nor did year 2 values differ based on year 1 infection status (Table 2).

**Table 2.** Characteristics of Adenovirus (AdV) 40/41 Diarrheal Episodes by Infection Group. Continuous Variables are Presented as Median (IQR), and Categorical Variables as n (%) of the Infection Group. Comparisons Between Year 1 (Y1) and Year 2 (Y2) Episodes Were Performed Using the Wilcoxon Rank-Sum Test, and Comparisons Across the Three Infection Groups Were Performed Using the Kruskal–Wallis Test. P Values for Categorical Variables Were Determined by the Chi-square or Fisher's Exact Test, as Appropriate. Y1+: Positive for AdV 40/41 Infection During Year 1 of Life; Y1-: Negative for AdV 40/41 Infection During Year 1 of Life; Y2+: Positive for AdV 40/41 Infection During Year 2 of Life; Y2-: Negative for AdV 40/41 Infection During Year 2 of Life; the Asterisks Indicate Two Missing Values From Y1 + Y1− AFe Group, Two Missing Values From Year 1 Y1 + Y1+ AFe Group and One From Year 2

…	Year 1 Episodes		Year 2 Episodes		*P V*alue
…	Y1 + Y2-	Y1 + Y2+	Y1-Y2+	Y1 + Y2+	
Days between Stool Collection to Year 1 Serum Sample	−132.5 (111)	−150 (96)	+101.5 (197)	+171.5 (152)	Y1: .31 Y2: .08
Day of Episode to Stool Specimen Collection	1 (1)	1 (2)	1.5 (2)	1 (2)	Y1: .64 Y2: .35
Episode Cycle Threshold Value	26.4 (15.2)	28.2 (12.2)	25.6 (14.6)	26.0 (15.1)	Y1 versus Y2: .22 Y1: .12 Y2: .65
Attributable Fraction	0.34 (0.54)*	0.32 (0.49)*	0.41 (0.51)	0.39 (0.50)*	Y1 versus Y2: 0.37 Y1: 0.22 Y2: 0.50
Duration of Diarrhea (d)	3 (3)	3 (3)	3 (3)	3 (2)	Y1 versus Y2: .11 Y1: .93 Y2: .45
Duration of Vomiting (d)	2 (5)	5.5 (7)	1 (3)	0 (1)	Y1 versus Y2: .12 Y1: .21 Y2: .14
RUUSKA Score	9 (4)	9 (4)	8 (4)	7.5 (2)	Y1 versus Y2: .05 Y1: .56 Y2: .28
RUUSKA Infection Severity	…	…	…	…	Y1 versus Y2: .04 Y1: .70 Y2: .36
Mild	11 (37)	14 (47)	24 (80)	22 (73)	
Moderate	14 (47)	11 (37)	6 (20)	8 (27)	
Severe	5 (16)	5 (16)	0 (0)	0 (0)	
Proportion of children with more than one AdV 40/41 PCR positive episode within the y	12 (40)	13 (43)	7 (23)	7(23)	Y1 versus Y2: .32 Y1: .79 Y2: 1.0

**Figure 4.**
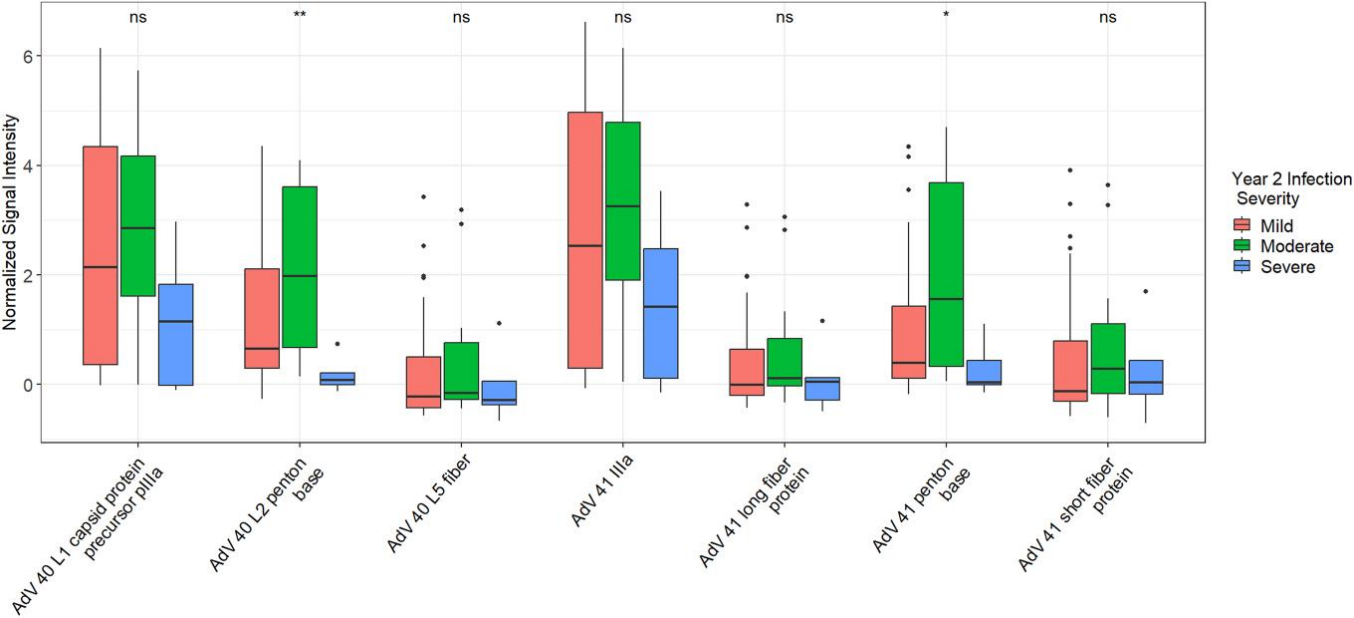
Box and whisker plots of top Adenovirus (AdV) 40 and 41 antigen reactivities in Principal Component (PC) 2 by year 2 infection severity. Mild, moderate, and severe infections are designated based on the Ruuska scoring system. NS = non-significant *P* value, * = *P* ≤ .05, and ** = *P* ≤ .01 by Kruskal–Wallis test.

Children with mild to moderate infections in year 2 had significantly higher reactivities to AdV 40 and 41 PB proteins at year 1 compared to those with severe infections (*P* = .008 and .032, respectively; Figure 4**).** Among the top AdV 40/41 antibody reactivities in PC2, AdV 41 PB was most strongly associated with a reduction in year 2 episode-specific attributable fraction (AFe) and an increase in year 2 cycle threshold (CT) value after adjusting for year 1 infection status (β = −0.14, *P* = .056, and β= 3.77, *P* = .11, respectively; Supplementary Figure 7), although neither association reached statistical significance. There was also a strong and significant correlation between 40 and 41 external antigen antibody reactivities, including PBs, long fibers, and short fibers, which had higher correlations compared to AdV 26, 4, and 5 on the array (Spearman's Correlation Coefficients 0.83, 0.98, 0.99, respectively, Supplementary Figure 8).

**Figure 5.**
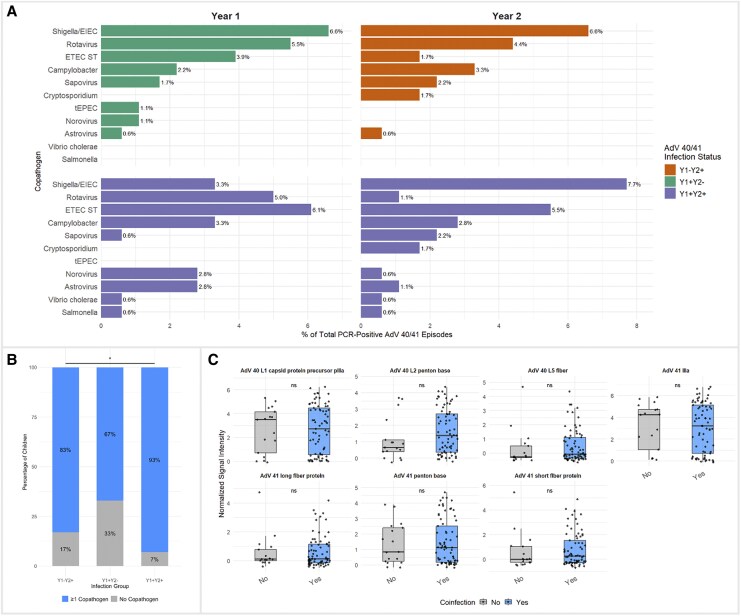
*A*, Distribution of AdV 40/41 copathogens by infection group. Bar plots show the proportion of different pathogens detected as coinfections with AdV 40/41 (defined as AdV PCR-positive episodes with the other pathogen having an attributable fraction estimate [AFe] ≥ 0.5). Proportions represent the percentage of that pathogen among all AdV PCR-positive infections over the two-year study period. *B*, Stacked bar plot of the proportion of children with ≥1 copathogen (blue) versus no copathogens (gray) in each infection group (Y1 + Y2-, Y1 + Y2+, Y1-Y2+). Percentages are calculated per group and displayed on each segment. A single asterisk indicates a statistically significant difference in coinfection prevalence across the three groups (Chi-squared test, *P* < .05). *C*, Boxplots of normalized signal intensity (SI) for top AdV 40/41 antigens, stratified by whether the child ever had a coinfection. Boxes show the interquartile range (IQR) with the median as a horizontal line; individual child antibody values are overlaid with jitter. Two-sided Wilcoxon rank-sum tests were used to compare “No” versus “Yes” coinfection groups, with significance indicated above each pair of boxes (ns = not significant).

AdV 40/41 coinfections were common, with top co-pathogens including *Shigella*, *Campylobacter*, Enterotoxigenic *Escherichia coli (*ETEC*)*, and Sapovirus (Figure 5*A*). The proportion of children with coinfections differed by AdV 40/41 infection group with the highest percentage of coinfections seen in children with year 2 reinfection (Figure 5*B*). Coinfection status did not significantly affect antibody levels for any top AdV 40/41 antigens (Figure 5*C*).

## DISCUSSION

Using a comprehensive multi-AdV microarray and serum samples from this well-characterized birth cohort of Bangladeshi infants, we saw that AdV PB IgG reactivity at year 1 was associated with protection from year 2 adenovirus 40/41 infection. We further saw an association between year 1 serum PB reactivity and reduced year 2 infection severity in those who had AdV 40/41 infection. These findings elevate PB as a promising antigen target for vaccine or monoclonal antibody production.

Though this data is limited by cohort sample size relative to the variable number assessed, PCA allowed us to reduce the number of variables we looked at, reducing the risk of false discovery. Additionally, close diarrheal surveillance in this cohort allowed for exposure groups to be delineated, which was reflected in the AdV 40/41 external proteins loaded into PC2. As hypothesized, key external structures unique to AdV 40 and 41 that allow for gastrointestinal tropism were most associated with a reduction in year 2 infection. AdV PB association with a reduced year 2 infection, reduced year 2 clinical severity, and reduced viral load (non-significantly) along with its biologic plausibility, makes it an optimal antigen for further study.

Mother and child anthropometrics and child sex differed among the four AdV 40/41 infection groups, consistent with known effects of malnutrition [25] and sex [26] on the adaptive immune response. Unexpectedly, children in households with private bathrooms were more likely to have year 2 AdV 40/41 infections than those with shared bathrooms, possibly reflecting selection bias, random variation, or differences in hygiene and behavior.

AdV 40/41 episodes in year 1 were more severe than those in year 2, though year 2 severity did not differ by prior year infection. While validation in larger cohorts is needed, this suggests that milder year 2 infections may reflect toddlers’ greater capacity to manage illness rather than robust infant immune responses. This is further supported by observed differences in T cell populations between children in high- and low-income countries [27] and, in the case of rotavirus, limited activation of antiviral CD4^+^ T cells during acute infection [28]. Thus, virus-specific T and B cell responses are important components to consider. This concept advocates for a prophylactic monoclonal antibody strategy [29] to provide protection during a period of maximal vulnerability in a population where robust and prolonged active immune response to vaccine antigen may be insufficient. Although systemic IgG showed protective potential here, future studies should evaluate antibody function assays and mucosal Ig responses.

Coinfections were common in this cohort, consistent with large multicenter studies, particularly for AdV 40/41 infections [4, 5]. The highest proportion of AdV 40/41 coinfections occurred in children with infections in both year 1 and 2, though coinfections did not impact year 1 antibody reactivity to key AdV 40/41 antigens, including AdV 41 PB, suggesting virus-specific humoral immune responses are relatively independent of concurrent enteric infections.

A challenge of this study was delineating the clinical significance of AdV 40/41 stool PCR positivity, particularly in children with coinfections and/or with multiple sequential positive AdV 40/41 stool. As AdV 40/41 has a relatively long pre- and post-symptomatic viral shedding duration, some children were positive for AdV 40/41 on multiple specimens surrounding an episode [30]. The episode chosen for analysis was the one with the lowest CT value to represent episodes with the highest viral burden and therefore most likely culprit of diarrheal disease. However, given the high sensitivity of TAC, which has been shown in multicenter studies to detect AdV 40/41 at up to five-fold higher frequencies than conventional enzyme immunoassays, a negative stool PCR suggests the absence of infection [4, 31, 32]. As these children had multiple stool samples obtained throughout both years, we assume that those we designated negative for infection in that year were unlikely to have experienced significant AdV 40/41 infection/exposure in that year.

We could not determine serotype-specific associations, as the TAC platform does not distinguish AdV 40 from 41. High correlations between AdV 40/41 external proteins suggest cross-reactivity or neutralization, as recently shown for species F antibodies targeting the short fiber [33]. Though the highest correlations were seen between 40 and 41 antigens, there were also high correlations between external structures and other AdVs on the array. This raises the possibility of cross-protection between F and non-F serogroups, particularly in the case of AdV 4, a component of the oral AdV vaccine used in the United States military populations [34, 35]. However, as this cohort lacks AdV respiratory infection data, this also indicates the potential for false-positive antibody reactivity to 40/41 antigens on the array. Future arrays can focus further on linear epitopes within the PB and fiber proteins for 40/41, as we have done previously for SARS-CoV-2 [36], which may separate reactivities from non-F groups. For instance, divergent regions from non-F groups include amino acid (aa) 230 to 300 of the AdV40/41 PB proteins, and aa 170–215, 260–320, and 340–390 of long fiber proteins [8, 16]. Despite these limitations, this study provides a first exploration of AdV 40/41 antibody responses in this population and highlights PB as a potential target for preventive strategies.

## Supplementary Material

jiaf558_Supplementary_Data

## Supplementary Data

Supplementary materials are available at *The Journal of Infectious Diseases* online (http://jid.oxfordjournals.org/). Supplementary materials consist of data provided by the author that are published to benefit the reader. The posted materials are not copyedited. The contents of all supplementary data are the sole responsibility of the authors. Questions or messages regarding errors should be addressed to the author.
