# Supplementary material for: Adenovirus 40 and 41 Antibodies Associated With Protection From Infection in a Bangladeshi Birth Cohort
Source: J Infect Dis. 2025 Nov 5;233(2):e430–40. doi: 10.1093/infdis/jiaf558 (PMC12776611; doi:10.1093/infdis/jiaf558)
Supplement: jiaf558_Supplementary_Data [file jiaf558_supplementary_data.docx]

**Adenovirus 40 and 41 antibodies associated with protection from infection in a Bangladeshi birth cohort- Supplementary Appendix**

Jennifer Hendrick^1*^, Jennie Z. Ma^2^, Vu Huynh^3^, Jozelyn V. Pablo^3^, Andy A. Teng^3^, Amit Oberai^3^, Joseph J. Campo^3^, David Camerini^3,4,5^, William A. Petri Jr.^1,6^

^1^Department of Medicine, University of Virginia Health System, Charlottesville, VA, USA;

^2^Department of Public Health Sciences, University of Virginia School of Medicine, Charlottesville, Virginia, USA;

^3^Antigen Discovery Incorporated (ADI), Irvine CA, USA;

^4^Center for Virus Research, University of California, Irvine, CA, USA;

^5^Department of Developmental and Cell Biology, University of California, Irvine, CA, USA;

^6^Department of Microbiology, Immunology and Cancer Biology, University of Virginia Health System, Charlottesville, VA, USA

*Corresponding author: Jennifer Hendrick, University of Virginia, 345 Crispell Drive, Charlottesville, Virginia 22908-1340, USA., Email: [jms7uq@uvahealth.org](mailto:jms7uq@uvahealth.org); Phone: 434-982-3950; ORCID ID 0000-0001-7352-2596.


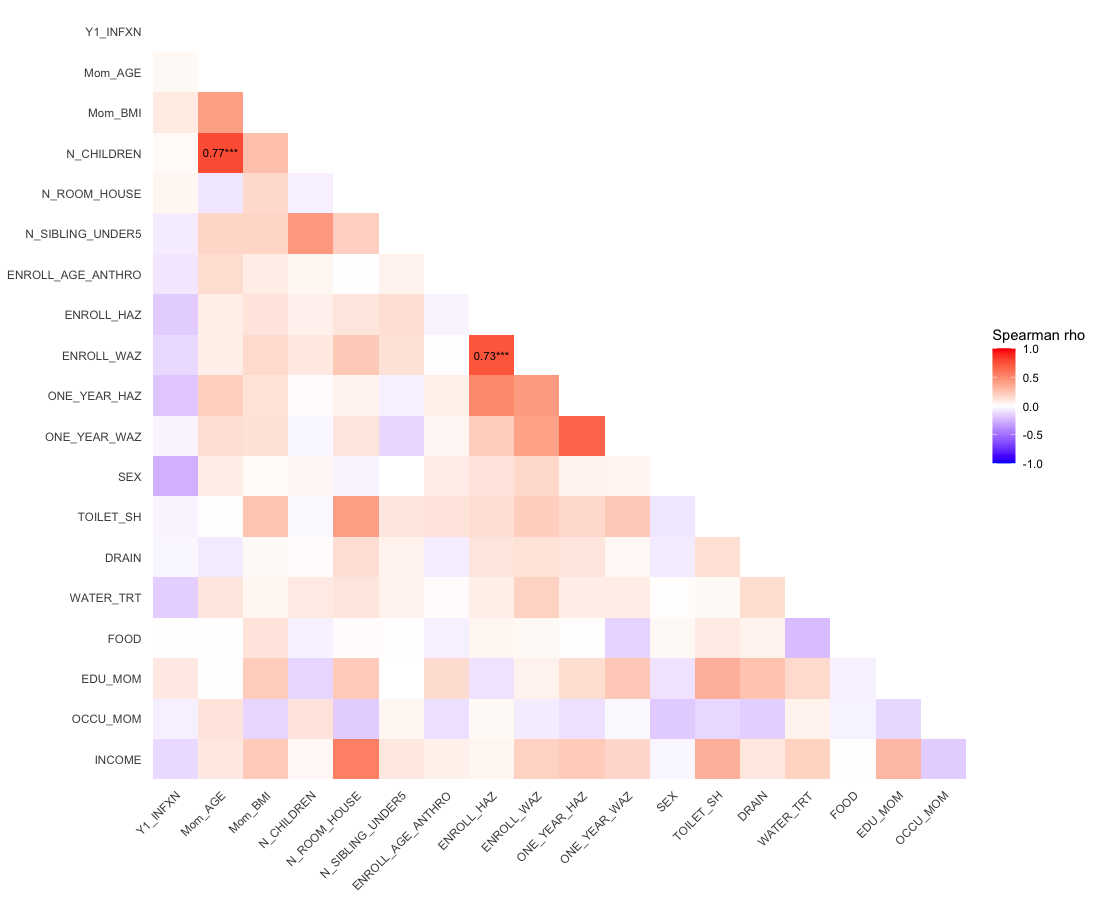


**Fig S1:** Lower triangle heatmap displaying pairwise Spearman correlation coefficients between Table 1 variables. Each tile represents the correlation (ρ) between two variables, with color indicating the strength and direction of the association (blue = negative, red = positive). Tiles are annotated with correlation coefficients where |ρ| > 0.7; asterisks denote statistical significance (* p < 0.05; ** p < 0.01; *** p < 0.001).


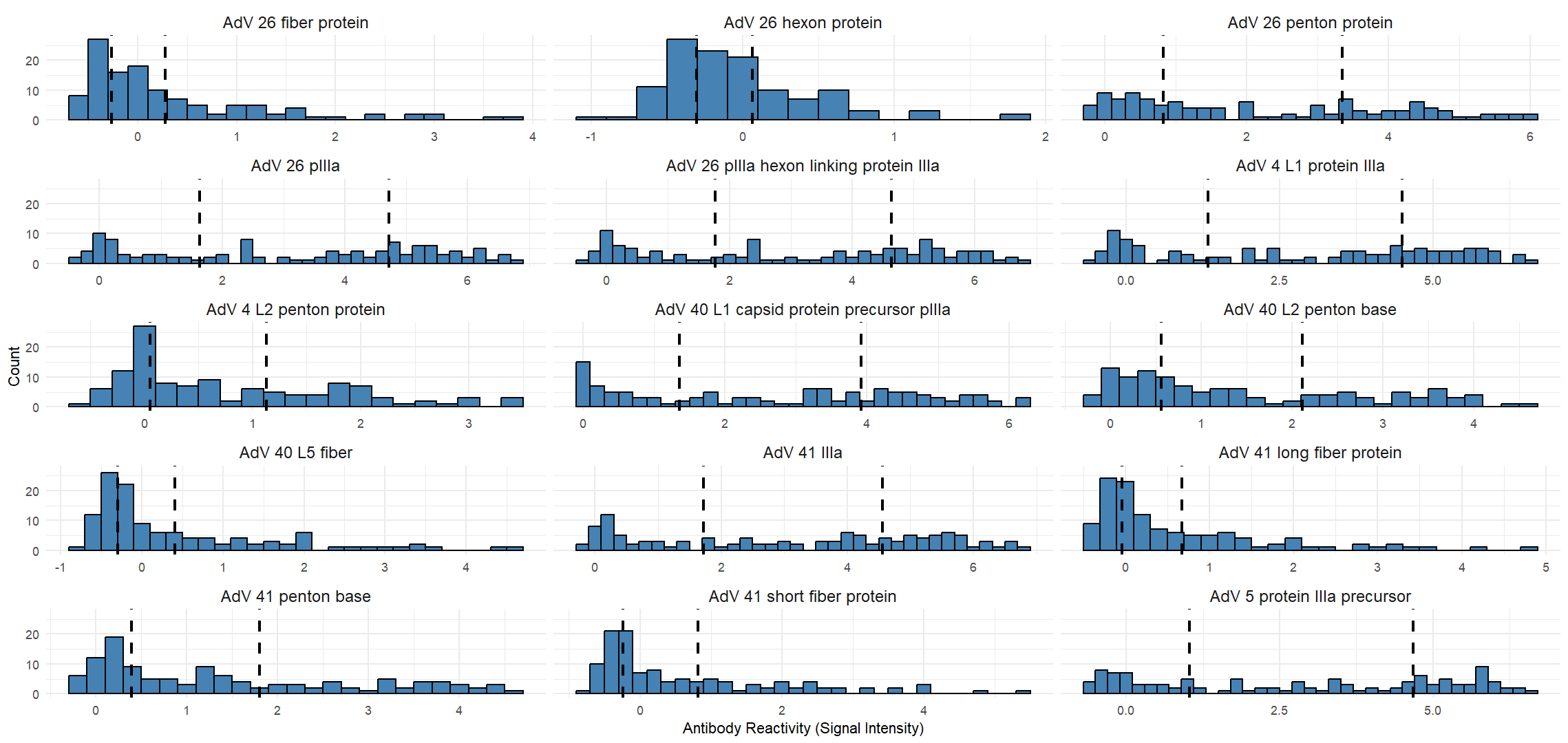


**Fig S2:** Histograms showing the distribution of antibody signal intensities for the top 15 PC2 antibody reactivities. Blue bars represent the frequency of antibody values by raw count. Blue dashed vertical lines indicate the tertile cut points (dividing the data into three equal groups) for each antigen.

**Table S1:** Leading principal components (PCs) from principal component analysis (PCA) , sorted by top antibody reactivities in PC2.

**
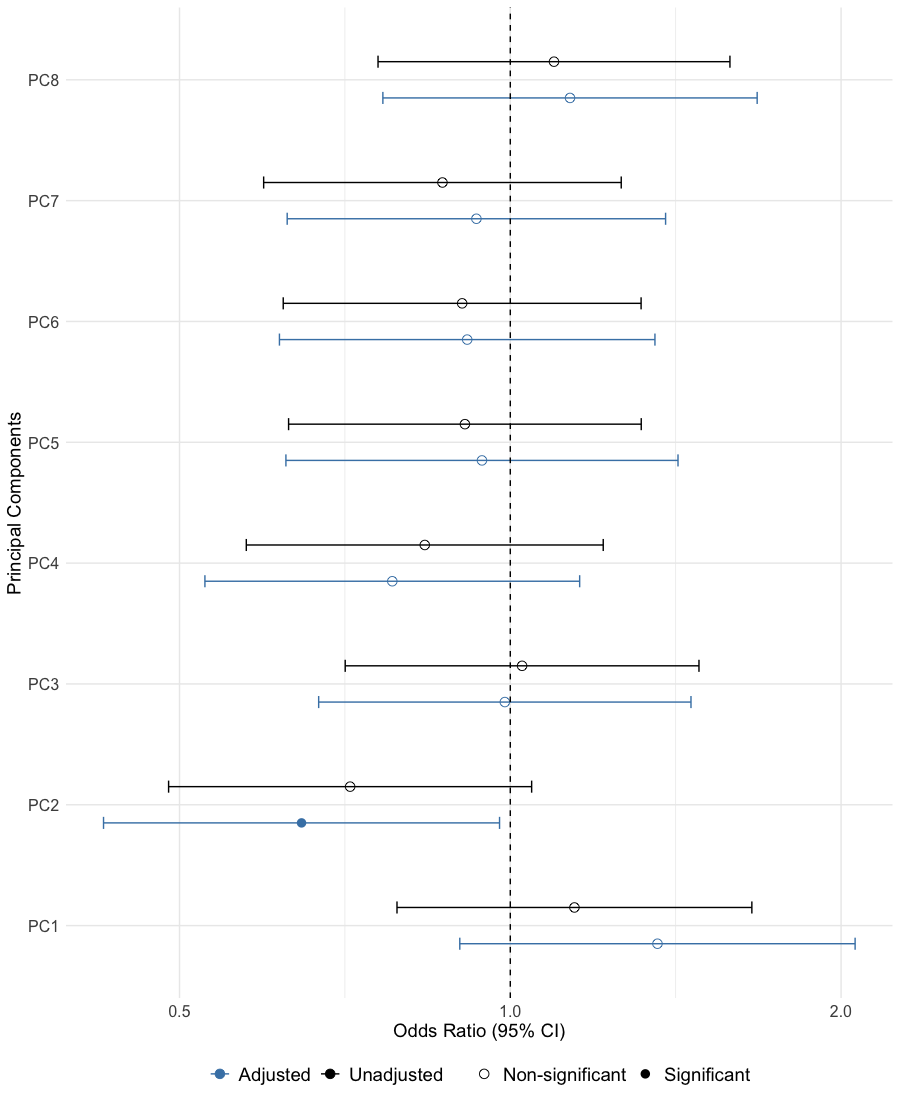
**

**Fig S3**: Forest plot showing odds ratios (ORs) and 95% confidence intervals (CIs) for the leading eight principal components (PCs) for their associations with year 2 infection, adjusted for year 1 infection. Both unadjusted (black) and adjusted (blue) models are shown; the adjusted model controls for relevant covariates (year 1 infection occurrence, sex, child enrollment HAZ, household shared toilet). Statistically significant associations (p < 0.05) have circle filled in, and non-significant associations are unfilled circles. Each point represents the estimated OR for a given PC, with horizontal lines indicating the 95% CI.

**
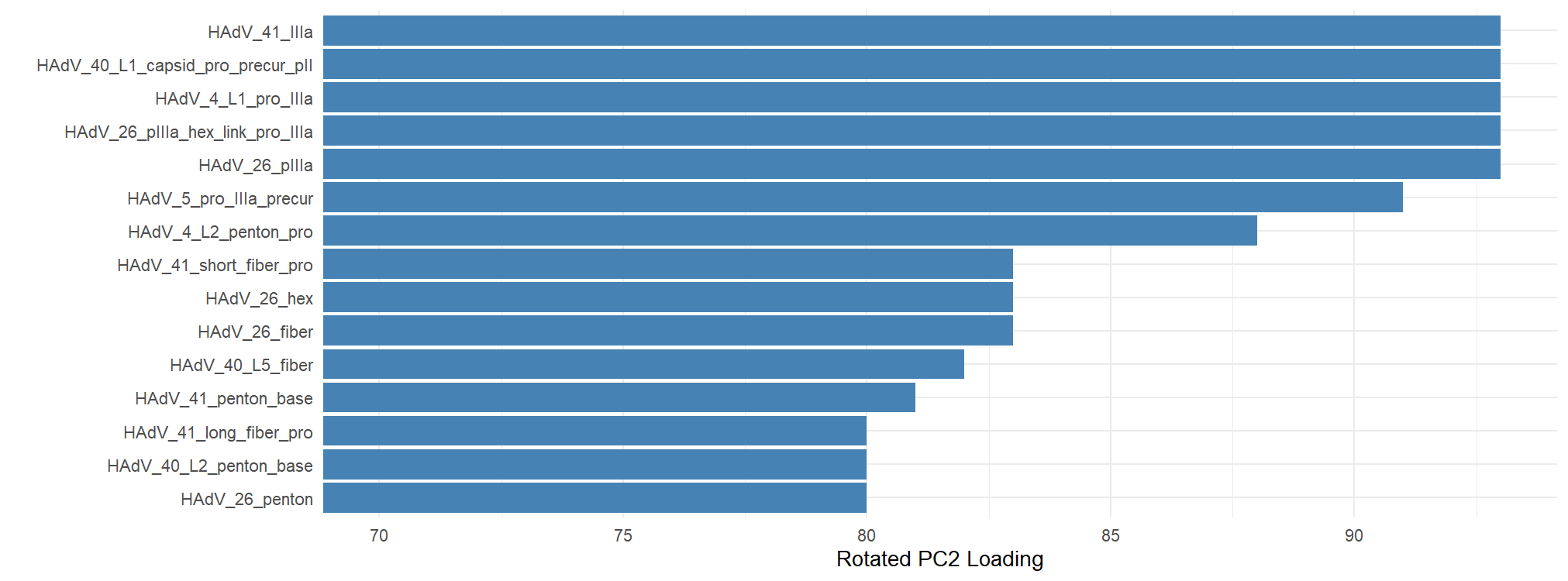
Fig S4:** Top 15 adenovirus antigens by their rotated loading scores on the second principal component (PC2), derived from a Varimax rotation of PCA performed on ranked antibody reactivities. Rotated loadings represent the contribution of each antigen to the underlying structure of the principal component, highlighting key antigens influencing variation in antibody responses.


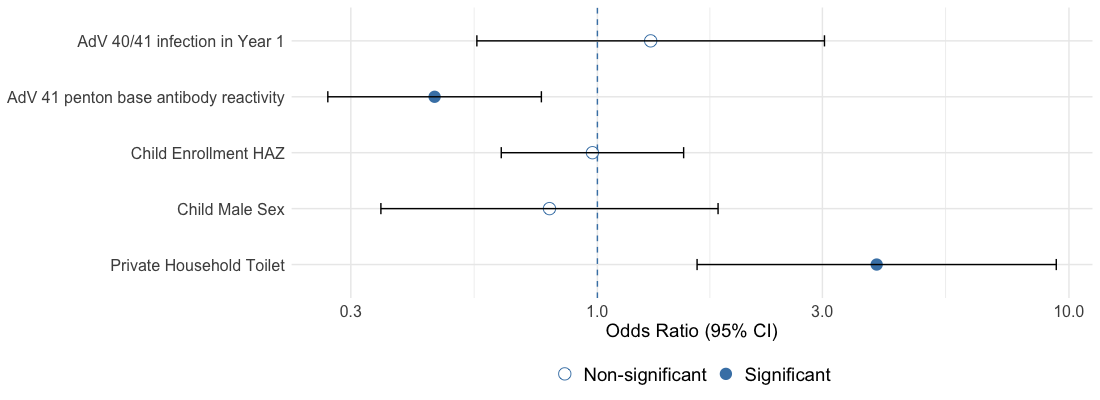


**Fig S5:** Forest plot showing adjusted odds ratios (ORs) with 95% confidence intervals for AdV 41 penton base association with year 2 infection and model covariates. The x-axis is on a logarithmic scale representing the OR values. Points and horizontal error bars indicate the OR estimates and their confidence intervals, respectively. Variables with statistically significant associations (p < 0.05) are filled in with a blue circle while non-significant variables are unfilled circles.


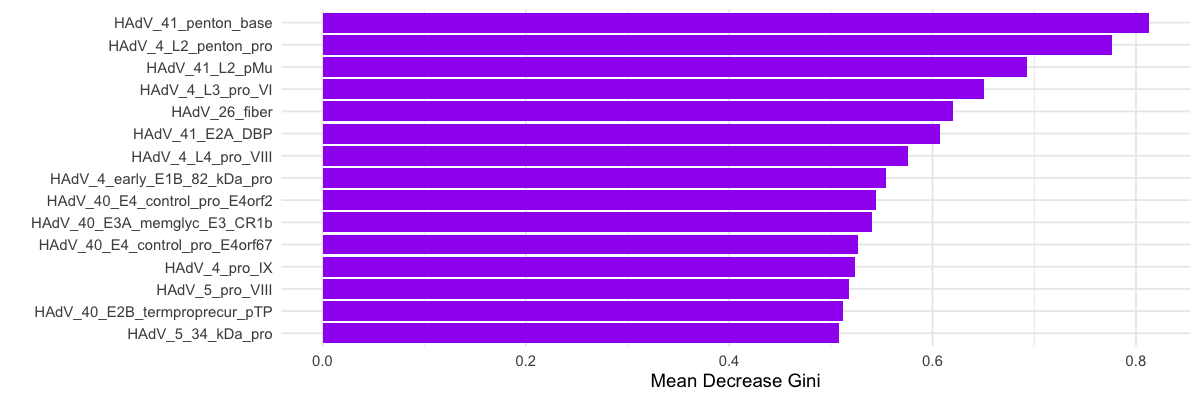
**Fig S6:** Variable importance plot from random forest analysis assessing top 15 antibody reactivities associated with year 2 AdV 40/41 infection. A mean decrease Gini quantifies how much a variable contributes to node homogeneity reduction.

**
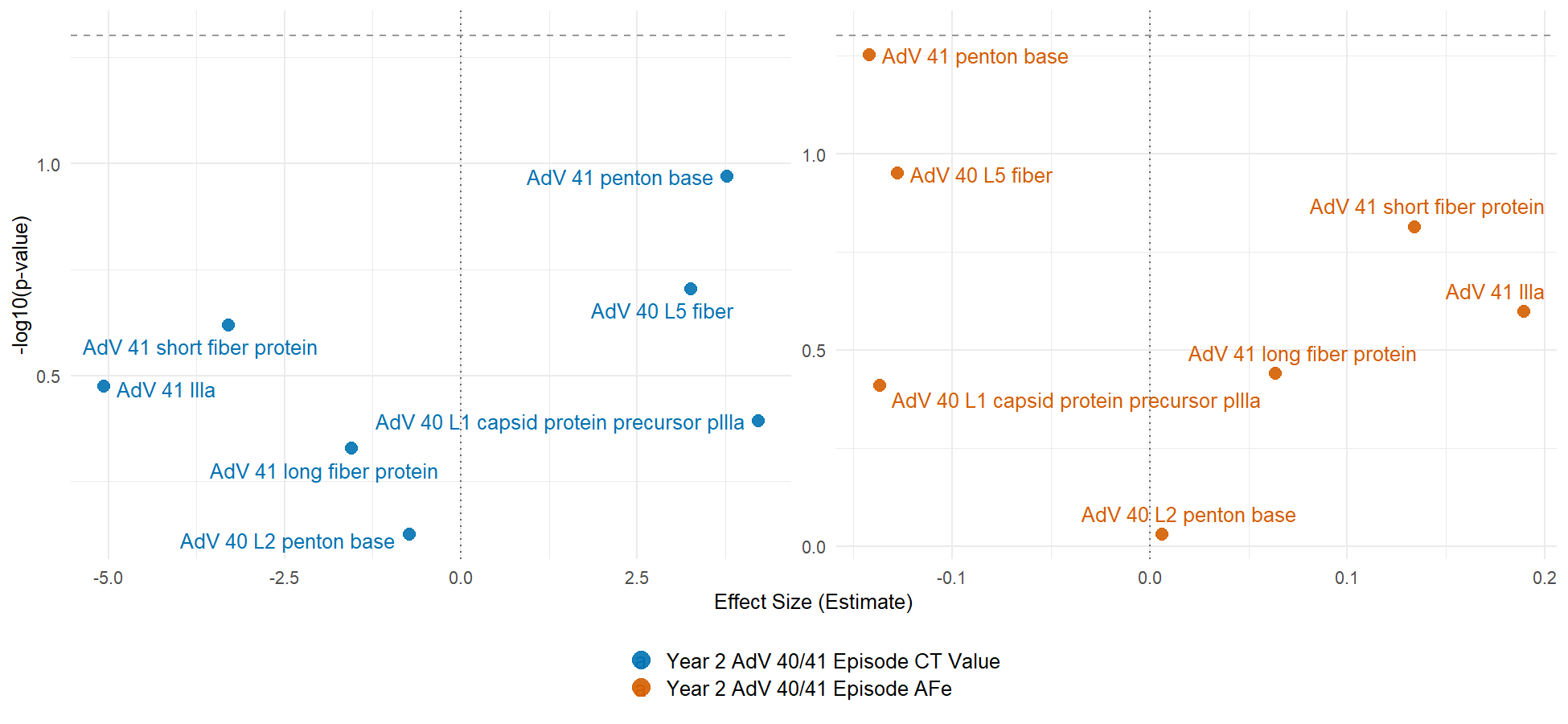
Fig S7:** Volcano plots showing the relationship between top AdV 40/41 antibody reactivities and year 2 infection burden, measured by AdV 40/41 episode cycle threshold (CT) value (blue, left) and AdV 40/41 episode Attributable Fraction (AFe) (orange, right). Each point represents 1 year antibody reactivity with the x-axis showing the estimated effect size from regression models and the y-axis showing statistical significance as −log10(p-value). The dashed horizontal line indicates the nominal significance threshold (*p* = 0.05).

**
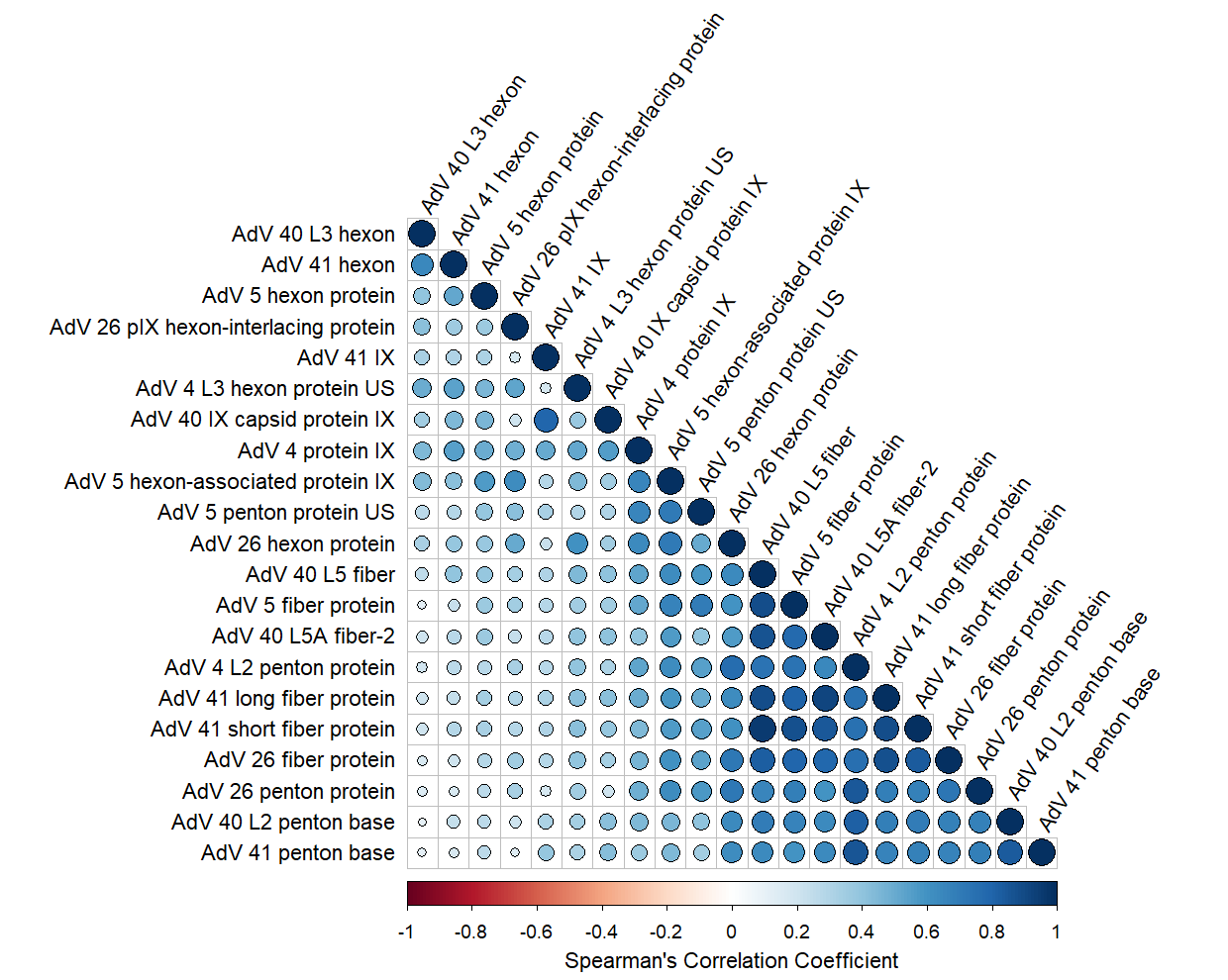
Fig S8:** Spearman **c**orrelation between Adenovirus (AdV) external protein reactivities from the multi- AdV array. The area of each circle is proportional to the absolute value of the correlation coefficient. The color of each circle, as per the heatmap on the bottom, represents both the direction and strength of the correlation.
